# Allergic Status, Long COVID, and Post-Restriction Respiratory Outcomes in Children: A Single-Center Questionnaire-Based Study

**DOI:** 10.3390/jcm15103982

**Published:** 2026-05-21

**Authors:** Giulia Brindisi, Alessandra Gori, Elia Pignataro, Giorgio Colletti, Sonia Iavarone, Alberto Spalice, Caterina Anania, Anna Maria Zicari

**Affiliations:** Department of Maternal, Infantile and Urological Sciences, La Sapienza University of Rome, 00161 Rome, Italy; alessandra.gori85@gmail.com (A.G.); elia.pignataro@uniroma1.it (E.P.); giorgio.colletti@uniroma1.it (G.C.); sonia.iavarone@uniroma1.it (S.I.); alberto.spalice@uniroma1.it (A.S.); caterina.anania@uniroma1.it (C.A.); annamaria.zicari@uniroma1.it (A.M.Z.)

**Keywords:** long COVID, pediatric COVID-19, allergic rhinitis, asthma, SARS-CoV-2 vaccination, *Streptococcus pyogenes*, *Group A Streptocossus* (GAS), viral pharyngitis, post-pandemic respiratory infections, immune dysregulation

## Abstract

**Background:** The relationship between allergic status, SARS-CoV-2 infection, Long COVID, and post-restriction respiratory outcomes in children remains incompletely understood. This study aimed to explore the associations between allergic status and Long COVID, as well as between SARS-CoV-2 vaccination and post-restriction changes in allergic rhinitis (AR), asthma, and upper respiratory infections, in a pediatric tertiary-care cohort. **Methods:** We conducted a single-center, questionnaire-based observational study involving children aged 0–16 years, who were followed at the Pediatric Allergy Clinic of Umberto I Hospital in Rome. Parents completed an email-based questionnaire addressing SARS-CoV-2 infection, vaccination, persistent post-infectious symptoms, allergic diseases, and respiratory infections following restrictions. Analyses of Long COVID were limited to children with confirmed SARS-CoV-2 infection. **Results:** A total of 214 questionnaires were analyzed. Allergic status was not significantly associated with SARS-CoV-2 infection in the overall cohort. Among infected children, allergic status was independently associated with higher odds of Long COVID (adjusted OR 3.12, 95% CI 1.20–8.09; *p* = 0.019). Severe acute infection was also strongly associated with Long COVID (adjusted OR 6.84, 95% CI 2.72–17.21; *p* < 0.001). Complete vaccination was associated with lower odds of SARS-CoV-2 infection in the overall sample (adjusted OR 0.20, 95% CI 0.09–0.46; *p* < 0.001) but was not independently associated with Long COVID among infected children. After the removal of COVID-19 restrictions, 90.1% of allergic children reported worsening AR and 52.0% reported worsening asthma, with no significant association with SARS-CoV-2 infection or Long COVID. *Group A Streptocossus* (GAS) pharyngitis was reported in 50.0% and viral pharyngitis in 10.7% of the cohort, with no significant differences between allergic and non-allergic children. **Conclusions**: In this single-center, questionnaire-based pediatric cohort, allergic status was correlated with increased likelihood of Long COVID among children with confirmed SARS-CoV-2 infection; however, it was not associated with a higher risk of infection itself. Complete vaccination was linked to a reduced risk of infection, whereas no independent correlation with Long COVID was identified. Post-restriction exacerbation of allergic respiratory symptoms was prevalent, while the incidence of bacterial and viral pharyngitis did not vary significantly according to allergic status.

## 1. Introduction

The long-term consequences of severe acute respiratory syndrome coronavirus 2 (SARS-CoV-2) infection in pediatric populations remain an active area of investigation. The SARS-CoV-2 pandemic has impacted global human health, with over 777 million confirmed cases and approximately 7.1 million fatalities reported as of the end of February 2026 [1].

In children, symptoms of SARS-CoV-2 infection were predominantly mild or absent, and its clinical presentation frequently overlapped with chronic conditions such as allergies, presenting significant challenges for differential diagnosis [2,3].

Notably well-managed allergic conditions do not appear to elevate susceptibility to SARS-CoV-2 infection; in contrast, emerging evidence indicates that controlled allergic symptoms may serve as a protective factor against COVID-19 [4,5,6].

To date, SARS-CoV-2 infection has resulted in numerous alterations and repercussions on both mental and physical health, particularly among susceptible populations such as children. This has prompted the conceptual differentiation between the pre- and post-COVID-19 eras [7,8].

COVID-19 is not solely an acute illness but may also result in a spectrum of long-term symptoms, known as Long COVID.

The World Health Organization (WHO) defines Long COVID as a condition occurring in individuals with a history of confirmed SARS-CoV-2 infection, typically 3 months after disease onset, with symptoms lasting at least 2 months and not attributable to alternative diagnoses [9].

Pediatric Long COVID is increasingly acknowledged as a clinically significant condition, although its incidence in children remains lower compared to adults. Studies involving pediatric populations have indicated that persistent symptoms are present in approximately 4–15% of infected children [10,11].

This complex multisystem disorder affects public health and involves all organs to varying degrees, with persistent symptoms such as fatigue, headache, sleep disturbances, and concentration problems [11,12,13].

It may be associated with various risk factors, including demographic and clinical characteristics, the presence of allergies, COVID-19 vaccination, microbiome dysbiosis and SARS-CoV-2 variants [13].

From an immunopathological perspective, Long COVID has been linked to ongoing immune dysregulation in certain studies, although the precise mechanisms underlying this phenomenon remain only partially understood. Indeed, persistent immune activation, difficulty overcoming inflammation, and impaired immune cell function are integral factors in its pathogenesis.

**Figure 1:**
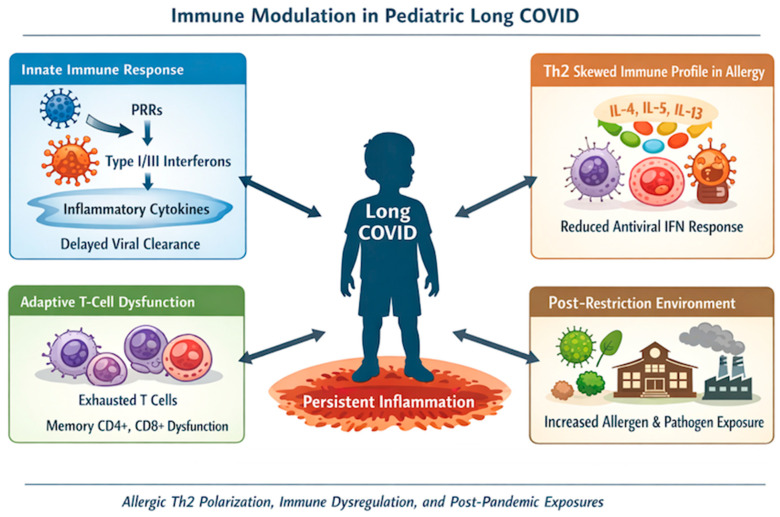
Hypothetical immune pathways potentially involved in pediatric Long COVID.

Specifically, defective early innate antiviral responses, notably diminished type I/III interferon signaling, may impede viral control and foster chronic immune activation. In this regard, Figure 1 illustrates a hypothetical, literature-supported conceptual framework that summarizes biologically plausible immune pathways potentially contributing to pediatric Long COVID [14,15].

To date, the immunological determinants underlying susceptibility to Long COVID remain poorly understood.

Although allergic diseases do not appear to increase susceptibility to SARS-CoV-2 infection when adequately controlled [6], it is still unclear whether allergic inflammation can influence the persistence of symptoms after acute infection.

In this context, a Th2-skewed immune profile, increased ILC2 activity, and eosinophilic inflammation may represent biologically plausible host factors associated with altered post-infectious immune recovery rather than established causal mechanisms [15,16].

Furthermore, innate and adaptive immune alterations induced by SARS-CoV-2 may contribute to persistent symptoms through prolonged inflammatory signaling and disrupted immune homeostasis [14].

Together, these mechanisms may provide a literature-based framework for interpreting possible links between pre-existing immune polarization and Long COVID; however, they remain hypothetical in the absence of direct immunological assessment. Additionally, the removal of public health restrictions has been associated with increased circulation of respiratory pathogens and renewed environmental exposure to allergens [17,18].

This figure summarizes literature-based, biologically plausible mechanisms and does not represent pathways directly assessed in the present study.

Despite these considerations, the association between allergic status and Long COVID in pediatric populations remains inadequately explored, especially within the context of vaccination and the post-pandemic setting. Additionally, it is crucial to evaluate the impact of the removal of restrictions on the management of allergic symptoms and the incidence of bacterial and viral pharyngitis among children.

To investigate the various significant aspects defining the post-pandemic era, we have conducted a comprehensive survey that examined the following points, with the objective of exploring:

(a) The correlation between allergic status and Long COVID;
(b) The relationship between SARS-CoV-2 vaccination and Long COVID;
(c) The impact of the removal of restrictions on AR and asthma symptoms;
(d) The role of bacteria, specifically *Streptococcus pyogenes*, *Group A Streptocossus* (GAS), and viral pharyngitis in the post-pandemic era, among both allergic and non-allergic children.

## 2. Materials and Methods

### 2.1. Study Design

From late 2022 to early 2023, we conducted a single-centre, questionnaire-based observational study involving children aged 0 to 16 years, both allergic and non-allergic, who were under follow-up at the Pediatric Allergy and Immunology Clinic of Umberto I Hospital in Rome.

A structured, study-specific questionnaire was disseminated via electronic mail to the parents of enrolled children.

The complete questionnaire is included in the Supplementary Materials (Supplementary File S1).

Each completed questionnaire pertained to a single child and retrospectively gathered information regarding previous SARS-CoV-2 infection, vaccination status, persistent symptoms, allergic diseases, and respiratory outcomes following restrictions.

The questionnaire comprised 22 multiple-choice items encompassing the following domains: demographic and anthropometric characteristics; SARS-CoV-2 infection history and associated symptoms; vaccination status; potential Long COVID and related symptoms; allergic status, type of allergic disorder, and sensitization profile; post-restriction exacerbation or improvement of allergic symptoms; and post-restriction bacterial or viral pharyngitis.

For the purposes of this study, Long COVID was defined as the presence of at least one parent-reported persistent physical symptom lasting >12 weeks and a confirmed SARS-CoV-2 infection, not otherwise explained by the available questionnaire information [9].

BMI was analyzed by clinical categories recorded in the database (normal weight, overweight, risk of obesity, and obesity), according to NCHS guidelines [19].

COVID-19 severity was determined according to the symptom burden, with severe infection classified when six or more symptoms were reported.

This classification serves as a data-driven proxy for severity, relying on symptom burden rather than a standardized clinical definition.

GAS pharyngitis was characterized by at least one documented episode of *Streptococcus pyogenes* pharyngitis following restriction, predominantly confirmed through throat-swab analysis when available. Conversely, viral pharyngitis was characterized by parental reports of post-restriction viral episodes, as recorded in the questionnaire.

The questionnaire took approximately 10 min to complete. A total of 250 questionnaires were distributed, resulting in 214 analyzable responses. Anonymous data were collected via Google Forms and exported in Excel format for statistical analysis. The retrospective questionnaire design did not permit full temporal reconstruction of all infection-vaccination sequences or prospective clinical adjudication of persistent symptoms. Approval for the study was granted by the Ethics Committee of Sapienza University of Rome. Written informed consent was obtained from all enrolled participants.

### 2.2. Statistical Analysis

Model calibration was assessed employing the Hosmer–Lemeshow test, thereby affirming an enhanced model fit (refer to Supplementary Table S2).

Categorical variables were summarized as absolute frequencies and percentages. Comparisons between groups were performed using Pearson’s chi-square test or Fisher’s exact test, as appropriate.

To explore independent associations with specific outcomes, multivariable logistic regression models were constructed. For the evaluation of Long COVID, analyses were restricted to subjects with confirmed SARS-CoV-2 infection (COVID = 1), given the structural impossibility of Long COVID in non-infected individuals. Covariates were selected *a priori* on clinical grounds and included allergic status, sex, age range, BMI category, vaccination status, and infection severity. Adjusted odds ratios (aORs) with 95% confidence intervals were reported.

Additional exploratory logistic regression models were conducted to assess determinants of post-restriction worsening of allergic rhinitis and asthma among allergic children. The post-pandemic occurrence of group A beta-hemolytic streptococcal pharyngitis (GAS) and viral pharyngitis was compared between allergic and non-allergic subjects using univariate analysis and multivariable logistic regression adjusted for age and sex. A multinomial logistic regression model was fitted to compare no pharyngitis, GAS, and viral pharyngitis as distinct outcomes.

All tests were two-sided, and a *p*-value of less than 0.05 was regarded as statistically significant in this exploratory analysis. Statistical analyses were conducted utilizing IBM SPSS statistics version 27.

## 3. Results

### 3.1. Characteristics of the Study Population

A total of 214 children aged 0–16 years were enrolled, comprising 105 males (49.1%; mean age 9.13 ± 4.00 years) and 109 females (50.9%; mean age 8.74 ± 3.34 years). Participants were categorized into three age groups: 0–5 years (30.4%), 6–10 years (35.0%), and 11–16 years (34.6%).

Anthropometric characteristics by gender are summarized in Table 1.

Allergic diseases were documented in 71.0% of the cohort (152 of 214). Confirmed SARS-CoV-2 infection was observed in 66.4% of children (142 of 214); 48.1% of patients (103 of 214) underwent a complete vaccination course. The distribution of allergies, infections, and vaccinations across age groups is shown in Table 2a,b.

**Figure 2:**
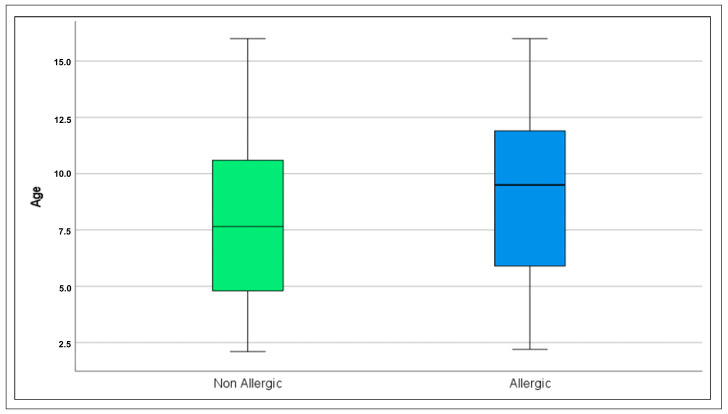
Age distribution according to allergic status. Boxes represent interquartile ranges; horizontal lines indicate medians.

The box plot depicted in Figure 2 offers a graphical representation of the age distribution stratified by allergic status. It confirms that the median age among allergic children is higher (median: Allergic 9.50 years versus Non-Allergic 7.65 years; *p* = 0.006).

Long COVID was identified in 25.2% of the overall sample (54 of 214) and in 38.0% of infected subjects (54 of 142). Among all the examined children, 40.2% suffered from allergic asthma, 65.4% from AR, and 37.4% from both asthma and AR. After the removal of COVID-19 restrictions, 54.2% of the entire cohort (116 of 214) reported becoming sicker; 107 had bacterial pharyngitis (GAS), and 23 had viral pharyngitis. GAS pharyngitis was documented in 50.0% of children (107 of 214), whereas viral pharyngitis occurred in 10.7% (23 of 214).

The distribution of AR and asthma, stratified by age, is detailed in Table 3.

### 3.2. Preliminary Analyses and Rationale for Population Restriction

Preliminary bivariate analyses were performed to examine the relationship between Long COVID and specific demographic and clinical variables, namely allergic status, gender, age group, BMI category, vaccination status, and severity of COVID-19. These variables were chosen a priori based on biological plausibility and existing literature indicating their potential impact on immune regulation, infection severity, and post-viral sequelae.

Initial descriptive analyses were performed on the entire cohort (N = 214) to offer an overview of distributional differences. However, since Long COVID can only manifest in individuals with confirmed SARS-CoV-2 infection, including uninfected children (COVID = 0) introduces a structural dependency, which may potentially result in biased associations mediated by infection status.

Subsequent analyses were limited to children with confirmed SARS-CoV-2 infection (*n* = 142), as Long COVID can only manifest within this subgroup. The following data pertain to a subset of the SARS-CoV-2-infected population (*n* = 142).

Within the infected subgroup, Long COVID was observed in 54 out of 142 subjects (38.0%).

**Figure 3:**
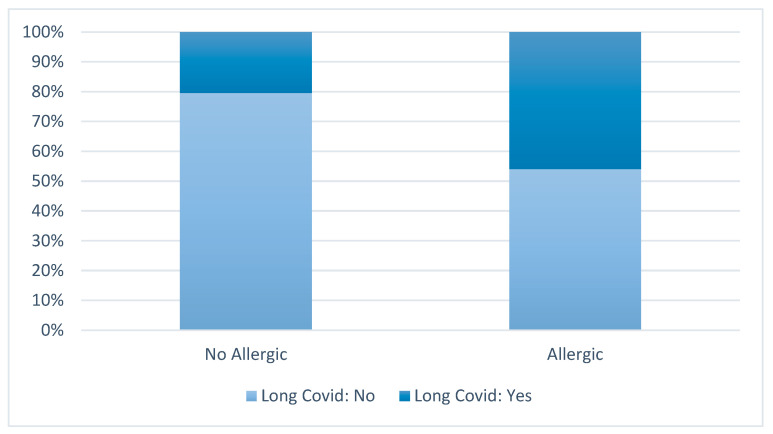
Prevalence of Long COVID among SARS-CoV-2-infected children according to allergic status. Analyses were restricted to children with confirmed SARS-CoV-2 infection.

The allergic status was significantly correlated with Long COVID (*p* = 0.0069), with allergic children demonstrating a markedly higher incidence of persistent symptoms compared to non-allergic subjects (45.9% versus 20.5%; see Figure 3). Within this cohort, Long COVID was identified in 38.0% of the participants. The association between allergic status and Long COVID was statistically significant, with a greater prevalence observed among allergic children relative to non-allergic children (45.9% versus 20.5%; *p* = 0.0069). Female sex was also associated with a higher prevalence of Long COVID (47.4% versus 27.3% in males; *p* = 0.022).

A notable association with gender was also identified (*p* = 0.022).

The female gender was associated with a higher prevalence of Long COVID (47.4% versus 27.3%).

The age range did not exhibit a statistically significant association (*p* = 0.342), and no apparent monotonic trend was observed across the three predefined age groups. Conversely, the BMI category was statistically associated with the distribution of Long COVID (*p* = 0.014), with increased frequencies identified among overweight and obese individuals. However, interpretation of the obesity subgroup is constrained by the limited sample size.

Among infected children, those who had completed the vaccination cycle exhibited a lower incidence of Long COVID compared to unvaccinated individuals (22.0% versus 47.2%). A statistically significant correlation was identified between complete vaccination status and the occurrence of Long COVID among COVID-positive subjects (χ^2^ = 12.05, *p* = 0.002).

Finally, the severity of infection demonstrated the most significant correlation (*p* < 0.001), with a distinct gradient: Long COVID was observed in 71.4% of severe cases, compared to 24.0% of non-severe cases.

Therefore, exclusively in subjects that are infected, we observe that:

- Allergy, gender, BMI, vaccination, and severity are associated with Long COVID
- Age is not associated.

Table 4 summarizes the preliminary analysis described above.

### 3.3. Association Between Long COVID and Allergic Status: Multivariable Analysis

In the univariate analysis restricted to children who tested positive for SARS-CoV-2, the prevalence of Long COVID was markedly higher among allergic children compared to non-allergic children (45.9% versus 20.5%; χ^2^ *p* = 0.0069). The crude logistic regression model corroborated this association (OR = 3.30; 95% CI 1.39–7.82; *p* = 0.007), as demonstrated in Table 5.

To account for measured confounders and evaluate independent associations with Long COVID, a multivariable logistic regression model was constructed incorporating allergic status, gender, age group, BMI category, vaccination status, and severity of infection. Following adjustment, allergic status remained independently associated with Long COVID (adjusted OR 3.12; 95% CI 1.20–8.09; *p* = 0.019). Infection severity was also significantly correlated with persistent symptoms (adjusted OR 6.84; 95% CI 2.72–17.21; *p* < 0.001). Although completion of vaccination was initially associated with a lower crude prevalence of Long COVID, this association was mitigated and no longer achieved statistical significance after adjustment for disease severity and other measured covariates (adjusted OR 0.54; 95% CI 0.16–1.83; *p* = 0.323).

Although female sex and higher BMI categories were associated with increased crude odds, these associations were attenuated after multivariable adjustment. Age range did not demonstrate a statistically significant effect in either crude or adjusted analyses.

Additional sensitivity analyses reaffirmed the robustness of the association between allergic status and Long COVID across various alternative outcome definitions and model specifications. Detailed results are provided in Supplementary Table S1.

The exclusion of respiratory-overlapping symptoms—including rhinitis, cough, anosmia, and exertional dyspnea—did not significantly alter the prevalence of Long COVID among infected children, which remained at 38.0%. This indicates that persistent symptoms were not solely attributable to allergic or respiratory manifestations.

Utilizing a more conservative definition that excluded additional non-specific symptoms, it was observed that allergic status continued to be independently associated with Long COVID (adjusted OR 3.84, 95% CI 1.30–11.36; *p* = 0.015).

Moreover, recoding BMI as normal weight versus excess weight enhanced model stability, with excess weight maintaining a significant association with Long COVID (adjusted OR 4.24, 95% CI 1.64–10.98; *p* = 0.0030). The simplified vaccination coding did not demonstrate an independent association with Long COVID after adjustment. However, the association between allergic status and Long COVID persisted after adjusting for age and in age-stratified exploratory analyses.

In this case, the multivariable model demonstrated a moderate degree of explanatory power (McFadden’s pseudo-R^2^ = 0.368) and exhibits strong discriminative ability (AUC = 0.883; 95% confidence interval, 0.819–0.934). Considering the observational nature of the study and the limited sample size of certain subgroups, these measures of performance should be regarded as descriptive rather than as definitive validation of the model.

**Figure 4:**
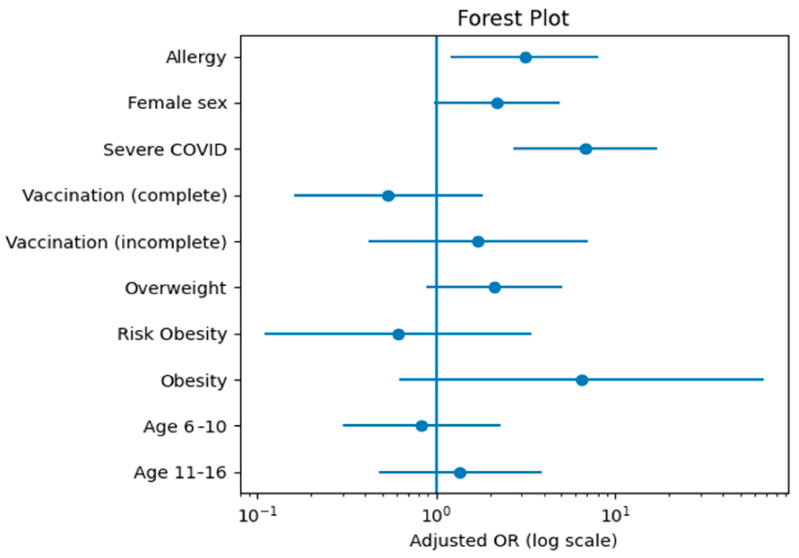
Adjusted odds ratios for factors associated with Long COVID among SARS-CoV-2-infected children. Odds ratios were derived from the multivariable logistic regression model including allergic status, sex, age group, BMI category, vaccination status, and COVID-19 severity. Horizontal bars indicate 95% confidence intervals; the x-axis is displayed on a logarithmic scale.

Figure 4 presents the adjusted odds ratios derived from the multivariable logistic regression model, depicted on a logarithmic scale to improve the interpretability of effect sizes and confidence intervals.

### 3.4. Vaccination, SARS-CoV-2 Infection, and Long COVID Among Allergic and Non-Allergic Groups

Our initial analysis evaluated whether allergic status was correlated with SARS-CoV-2 infection. No statistically significant association was identified (χ^2^ = 0.566, *p* = 0.452), and the odds of infection among allergic children did not differ significantly from those of non-allergic children (OR = 0.74, 95% CI 0.39–1.41). Within the entire cohort, complete vaccination was correlated with lower odds of SARS-CoV-2 infection (crude OR = 0.52; 95% CI 0.29–0.94; *p* = 0.029), and this correlation remained significant after adjustments for age and gender (adjusted OR = 0.20; 95% CI 0.09–0.46; *p* < 0.001). However, among children infected, complete vaccination status was not independently associated with Long COVID in multivariable models (adjusted OR = 0.54; 95% CI 0.16–1.83; *p* = 0.323). No statistically significant difference in the incidence of Long COVID was observed between vaccinated and unvaccinated individuals among the infected cohort.

To examine whether the effect of vaccination differed between children with and without allergies, initial stratified analyses were conducted.

In both cohorts, no statistically significant difference in the incidence of Long COVID was observed between vaccinated and unvaccinated infected children.

To formally assess effect modification, a logistic regression model was applied that included an interaction term between vaccination status and allergy status within the infected subgroup.

The interaction term was not statistically significant (*p* = 0.454), indicating no evidence that the association between vaccination status and Long COVID differed according to allergic status within the infected subgroup.

Overall, these findings indicate reduced likelihood of SARS-CoV-2 infection among fully vaccinated children within the overall sample; however, among infected children, no independent association between vaccination and Long COVID was observed in either allergic or non-allergic subjects.

Additional sensitivity analyses confirmed the robustness of the findings and the stability of results across multiple model specifications (see Supplementary Table S1).

Additional analyses limited to vaccine-eligible children (aged 5 years and above) confirmed that complete vaccination continues to be strongly associated with reduced odds of SARS-CoV-2 infection (adjusted OR 0.145, 95% CI 0.060–0.354; *p* < 0.001), thereby reinforcing the validity of the infection-related findings.

### 3.5. Worsening of Allergic Rhinitis and Asthma After Removal of COVID-19 Restrictions

Among allergic children (*n* = 152), a deterioration in allergic rhinitis (AR) following the relaxation of COVID-19 restrictions was observed in 90.1% of cases. Worsening of asthma was reported in 52.0% of allergic patients. An increase in the use of corticosteroids and bronchodilators was noted in approximately fifty percent of children with allergies. When analyzed according to SARS-CoV-2 infection status, no statistically significant differences in symptom exacerbation were identified (AR *p* = 0.072; asthma *p* = 0.120). Similarly, the aggravation of allergic symptoms was not significantly correlated with Long COVID among infected allergic children.

In pediatric patients with allergies, an exacerbation of symptoms is noted following the relaxation of restrictions; however, no statistically significant correlation is identified with either SARS-CoV-2 infection or the emergence of Long COVID. Multivariable logistic regression analyses, incorporating variables such as Long COVID status, vaccination status, COVID severity, age, BMI, and gender, failed to demonstrate any independent predictors for the aggravation of allergic rhinitis (AR) or asthma.

### 3.6. Bacterial (GAS) and Viral Pharyngitis in the Post-Restriction Era

GAS pharyngitis was identified in 50.0% of the cohort, whereas viral pharyngitis was observed in 10.7%. No statistically significant difference in GAS prevalence was observed between allergic and non-allergic children (58.1% vs. 46.7%; OR 0.63; *p* = 0.175).

Viral pharyngitis exhibited an inconclusive trend towards a higher prevalence among children with allergies (12.5% versus 6.5%; OR 2.07; *p* = 0.232).

In the multivariable logistic regression analysis, allergic status (adjusted for age range and gender) was not independently associated with GAS (adjusted OR = 0.68; *p* = 0.210) or viral pharyngitis (adjusted OR = 2.17; *p* = 0.182).

Age range and gender were also not significantly associated.

Interaction testing between allergic status and age did not reveal a significant effect modification.

To further evaluate potential differences in etiology, a multinomial logistic regression analysis was conducted, with “no pharyngitis” designated as the reference category. As delineated in Table 6, allergic status was not significantly correlated with either GAS or viral pharyngitis. No statistically significant interaction between allergic status and COVID status was identified. Furthermore, GAS was not associated with Long COVID among infected children (OR = 0.85; *p* = 0.760).

## 4. Discussion

This single-center, questionnaire-based investigation examined the correlation between allergic status and Long COVID, as well as post-restriction respiratory outcomes within a tertiary pediatric cohort. The principal findings are as follows: allergic status was not significantly associated with SARS-CoV-2 infection in the overall sample; however, among infected children, it was linked to increased odds of Long COVID after adjusting for measured covariates. Complete vaccination was associated with reduced odds of SARS-CoV-2 infection, whereas no independent association with Long COVID was observed among infected children. Worsening of allergic rhinitis (AR) and asthma following the relaxation of COVID-19 restrictions was frequently reported among allergic children, without a significant relationship to SARS-CoV-2 infection or Long COVID. Lastly, GAS and viral pharyngitis were commonly reported post-restriction outcomes but did not show significant differences based on allergic status. These findings should be interpreted considering the retrospective, questionnaire-based design and the tertiary-care setting of the cohort.

Given the multifaceted nature of this study, each finding is discussed separately below.

### 4.1. Long COVID Among Allergic and Non-Allergic Children

Within the subgroup of children with confirmed SARS-CoV-2 infection, allergic status was associated with increased odds of Long COVID, whereas no significant association was observed between allergic status and SARS-CoV-2 infection in the overall cohort. This pattern indicates that allergic status may be more pertinent to the persistence of post-infectious symptoms rather than to the occurrence of infection itself in this clinical context.

However, this interpretation should be approached with caution, as the study design does not permit causal inference, and the outcome was based on parent-reported symptoms rather than prospective clinical adjudication.

Importantly, supplementary sensitivity analyses specifically designed to mitigate symptom overlap with allergic respiratory disease reaffirmed the robustness of this association.

The prevalence of Long COVID remained unchanged after the exclusion of overlapping symptoms, and allergic status persisted as an independent factor associated with persistent symptoms across various model specifications. These findings indicate that the observed association is unlikely to be solely attributable to symptom misclassification or overlap with allergic diseases.

Our findings also correspond with prior research in both pediatric and adult populations, suggesting that female sex, elevated BMI, and increased severity of acute illness may be correlated with a greater burden of persistent symptoms [20,21].

Within our cohort, these variables demonstrated consistent directional relationships, albeit with some subgroup estimates constrained by sample size, thereby necessitating cautious interpretation. Consequently, these findings should be regarded as clinically pertinent associations rather than conclusive risk estimations.

The correlation between allergic status and Long COVID is biologically plausible; however, it should not be overinterpreted.

Type 2-mediated inflammation, altered interferon responses, mast-cell activation, epithelial barrier dysfunction, and prolonged immune activation have all been proposed as potential contributors to persistent post-viral symptoms [15,22,23,24,25].

However, all of these mechanisms may constitute biologically plausible hypotheses rather than mechanisms directly demonstrated by the present study.

Given that pediatric Long COVID has been linked to immune dysregulation, persistent inflammatory signaling, and altered adaptive responses, it has been hypothesized that a preexisting shift toward the Th2 response may be associated with altered post-viral immune resolution.

Furthermore, recent pediatric immunology studies have indicated that children experiencing persistent post-COVID symptoms may display alterations in T-cell function and cytokine production associated with low-grade, ongoing inflammation [15,22].

Within this context, allergic inflammation may serve as a host background linked to altered post-infectious immune recovery, rather than representing a conclusively established causal mechanism [26,27].

Furthermore, mast cell activation plays a central role in allergic diseases, and it has been hypothesized that mast cells contribute to the pathophysiology of Long COVID [23].

The ongoing activation of these cells could be a fundamental factor in the manifestation of typical Long COVID symptoms, such as fatigue, neurocognitive impairments, and respiratory issues. Although definitive causal evidence remains scarce, it is plausible that mediator release mediated by mast cells may play a role in the persistence of multisystem symptoms [15,24,25].

Another plausible mechanism, as demonstrated in the literature, involves dysfunction of the epithelial barrier in allergic diseases. In fact, the epithelium of the allergic airway exhibits impaired tight junctions and increased permeability of these junctions, potentially facilitating prolonged immune activation following viral challenges [15,28].

Impaired barrier function can amplify inflammatory cascades following viral infection and sustain tissue-level immune activation. To date, emerging evidence of the persistence of SARS-CoV-2 antigen in various tissues, even several months after infection, supports the hypothesis that impaired immune resolution may contribute to the chronicity of COVID-19 symptoms [22,28,29].

Importantly, none of these pathways were directly investigated in our study.

We did not perform immunophenotyping, cytokine profiling, ACE2 expression analysis, mast-cell marker assessment, epithelial biomarker evaluation, or viral persistence studies.

Therefore, the mechanistic considerations presented here should be regarded as hypothesis-generating and based on existing literature rather than explanatory conclusions derived from our own data. Overall, our results suggest that, within this tertiary pediatric cohort, allergic status was associated with Long COVID among infected children but was not associated with higher odds of SARS-CoV-2 infection in the overall sample. This distinction is clinically relevant, but it requires confirmation in larger prospective cohorts with standardized Long COVID definitions and deeper clinical and immunological characterization.

### 4.2. Vaccination and Long COVID

Within the overall cohort, full vaccination was correlated with reduced odds of SARS-CoV-2 infection, aligning with substantial epidemiological evidence that supports the vaccine’s efficacy in preventing symptomatic infection [30,31,32,33].

Conversely, among children who were infected, vaccination was not independently linked to Long COVID after adjusting for disease severity and other measured covariates.

Rather than indicating conflicting results, this pattern implies that the simple correlation between vaccination and ongoing symptoms might be diminished upon accounting for variations in initial disease severity and other measured factors [30,31,32,33].

Considering the retrospective study design, the small sample size of the infected subgroup, and the incomplete temporal reconstruction of infection-vaccination sequences, these findings should not be construed as evidence opposing a potential biological effect of vaccination on post-acute outcomes. Rather, they demonstrate that within this cohort, there is a definitive association with infection risk, but the evidence remains insufficient to establish an independent link with Long COVID following infection.

### 4.3. Allergic Rhinitis and Asthma Symptoms After Removal of Restrictions

A considerable proportion of children with allergies reported a deterioration in allergic rhinitis (AR) and asthma subsequent to the relaxation of COVID-19 restrictions. This observation aligns with pediatric research indicating enhanced control of respiratory allergies during lockdown durations and a subsequent exacerbation of symptoms following renewed contact with aeroallergens, respiratory viruses, and routine social interactions [34,35]. Brindisi et al. reported improved AR and asthma control during lockdown; likely attributable to reduced exposure to environmental triggers and respiratory infections. The study also described decreased use of reliever medication, consistent with a decline in asthma exacerbations and AR flares. The authors emphasized the significance of maintaining controller therapies, including inhaled corticosteroids and biologics, to prevent disease progression. Overall, maintaining optimal control of allergic respiratory diseases appeared to be essential during a viral epidemic [6].

Similarly, Kuitunen et al. examined the effect of COVID-19 restrictions on pediatric respiratory infections, noting a significant decline in emergency department visits and hospital admissions during the lockdown period. Additionally, the study reported a concurrent reduction in asthma exacerbations within the same timeframe [34].

Similar findings have been observed in children with wheezing or asthma, who experienced clinical improvement during COVID-19 restrictions, followed by a resurgence of symptoms and increased utilization of healthcare services after the restrictions were lifted [6,34,35].

The concept of “immune debt” has been posited to elucidate this phenomenon, whereby diminished exposure to prevalent pathogens during lockdown periods may result in a transient decrease in immune stimulation at the population level, subsequently leading to an elevated susceptibility upon re-exposure [36,37].

In our cohort, the exacerbation of AR and asthma symptoms was not significantly correlated with either SARS-CoV-2 infection or Long COVID. This indicates that the post-restriction increase in respiratory symptom burden among allergic children may have been primarily attributable to renewed exposure to environmental allergens and respiratory pathogens, rather than to post-COVID sequelae. Nonetheless, as symptom exacerbation was evaluated through questionnaires rather than confirmed by objective functional assessments, this interpretation should be approached with caution.

### 4.4. Post-Pandemic Bacterial and Viral Pharyngitis

GAS and viral pharyngitis were frequently reported during the post-restriction period, consistent with the broader literature documenting a resurgence of respiratory infections following the relaxation of public health measures [38,39].

A large multinational cross-sectional study by Dokal K et al. found that young children (3–4 years of age) had reduced immunity to GAS following the implementation of non-pharmaceutical interventions (NPIs), which corresponded to a notable subsequent increase in invasive GAS infections in children after NPIs were lifted [40]. European surveillance data similarly confirmed increased post-pandemic GAS circulation among children, often with atypical seasonal patterns [41]. Furthermore, a French interrupted time-series analysis documented a resurgence in severe pediatric Group A Streptocossus (GAS) infections subsequent to the reopening of educational institutions and the resumption of social activities [39].

In our cohort, however, no significant differences emerged between allergic and non-allergic children in either univariate or adjusted analyses.

In detail, GAS pharyngitis exhibited a high prevalence among allergic children; however, this difference did not attain statistical significance. No substantial variations were observed between allergic and non-allergic children in acquiring this infection, and allergic status was not independently correlated with bacterial pharyngitis in multivariate analyses.

Similarly, viral pharyngitis was not significantly associated with allergic status in multinomial regression analysis, despite a comparable trend toward higher prevalence in allergic subjects.

These findings imply that, within this clinical cohort, the post-pandemic rise in respiratory infections signifies broader alterations in pathogen circulation rather than a particular susceptibility associated with allergic status.

As these outcomes were based on retrospective parental reporting and were analyzed as secondary exploratory endpoints, they should be interpreted with caution and not as microbiologically adjudicated incidence estimates.

### 4.5. Limitations and Strengths

Our study possesses several limitations that warrant explicit acknowledgment.

Firstly, the retrospective, questionnaire-based, and cross-sectional design does not permit causal inference.

Secondly, the cohort was assembled within a single tertiary Pediatric Allergy and Immunology Clinic; consequently, it is enriched for allergic conditions, which restricts external generalizability and may be indicative of referral patterns and symptom reporting specific to this clinical environment.

Third, the evaluation of Long COVID and post-restriction symptom exacerbation was conducted using parental questionnaires rather than through standardized prospective clinical assessments, thereby introducing potential biases such as recall bias, misclassification, and overlap with pre-existing allergic respiratory symptoms.

Fourth, the available data did not permit a comprehensive temporal reconstruction of all infection-vaccination sequences or direct attribution to specific viral variant periods. Finally, some subgroup analyses were conducted on small sample sizes, which may have diminished precision and led to less stable estimates.

Despite these limitations, the study also has relevant strengths.

To the best of our knowledge, this study is among the few pediatric investigations that examine multiple clinically interconnected post-pandemic domains within the same pediatric specialty cohort. These domains include allergic status, SARS-CoV-2 infection, Long COVID, post-restriction exacerbation of allergic respiratory symptoms, and upper respiratory infections. Furthermore, the analyses concerning Long COVID were appropriately limited to children with confirmed SARS-CoV-2 infection, thereby preventing structural misclassification of the outcome.

Nevertheless, the coherence of the primary findings across various sensitivity analyses reinforces the internal robustness of the observed associations.

Overall, the study addresses a clinically significant area in which pediatric evidence is limited and may contribute to the formulation of hypotheses for future prospective, multicenter, and mechanistically focused studies.

## 5. Conclusions

This single-centre, questionnaire-based study indicates that, within a tertiary pediatric allergy cohort, allergic status correlates with increased likelihood of Long COVID among children with confirmed SARS-CoV-2 infection. Conversely, no significant correlation was identified between allergic status and SARS-CoV-2 infection in the overall sample. Complete vaccination was associated with reduced odds of infection; however, no independent association with Long COVID was observed among infected children after adjusting for measured covariates.

An increase in allergic rhinitis and asthma symptoms following the relaxation of COVID-19 restrictions was frequently documented; however, it was not significantly correlated with SARS-CoV-2 infection or Long COVID. Likewise, no notable differences were identified between allergic and non-allergic children regarding reported GAS or viral pharyngitis during the post-restriction period.

These findings should be approached with caution considering the retrospective questionnaire-based nature of the study, the symptom-based identification of Long COVID, the potential for recall and misclassification biases, and the limited generalizability stemming from a single-centre tertiary-care cohort. Instead of establishing causal relationships, our results serve as an exploratory indication that allergic status might be pertinent to the persistence of post-infectious symptoms in children. To elucidate this association more clearly, larger prospective, multicenter studies employing standardized definitions of Long COVID and incorporating more detailed clinical and immunological phenotyping are required.

## Figures and Tables

**Table 1:** Anthropometric characteristics of the patients.

Characteristics	Mean ± SD	Median	IQR
Age (years)			
Male	9.13 ± 4.00	9.30	6.40
Female	8.74 ± 3.34	8.90	5.40
Weight (kg)			
Male	38.42 ± 16.81	39.60	29.50
Female	36.74 ± 14.31	39.50	22.80
Height (cm)			
Male	137.35 ± 25.94	141.00	41.30
Female	136.10 ± 21.93	141.00	33.00
BMI (kg/m^2^)			
Male	19.05 ± 2.58	19.30	4.10
Female	18.84 ± 2.50	18.90	4.10

BMI: body mass index.

**Table 2:** (**a**) Baseline characteristics of the study population (N = 214). (**b**) Distribution of vaccination status, SARS-CoV-2 infection and allergic status by age group.

(**a**)				
**Characteristic**			**Count**	**Percent**
Gender				
- Male			105	49.1%
- Female			109	50.9%
Age group				
- 0–5 years			65	30.4%
- 6–10 years			75	35.0%
- 11–16 years			74	34.6%
(**b**)				
Variable	0–5 years (N = 65)	6–10 years (N = 75)	11–16 years (N = 74)	
Complete vaccination	15 (23.1%)	46 (61.3%)	42 (56.8%)	
SARS-CoV-2 infection	30 (46.2%)	54 (72.0%)	58 (78.4%)	
Allergy	40 (61.5%)	52 (69.3%)	60 (81.1%)	

**Table 3:** AR and asthma stratified by age.

Allergic Disease	Count	Percent
AR	140	
0–5 years	32	49.2%
6–10 years	50	66.7%
11–16 years	58	78.4%
Asthma	86	
0–5 years	19	29.2%
6–10 years	26	34.7%
11–16 years	41	55.4%

AR: allergic rhinitis.

**Table 4:** Bivariate association between Long COVID and selected variables among SARS-CoV-2-infected children (COVID = 1; *n* = 142).

Variable	Category	Long COVID Absent		Long COVID Present		χ^2^ (df)	*p*-Value
		N	%	N	%		
Allergy	Non-allergic	35	79.5%	9	20.5%	7.31 (1)	0.0069
	Allergic	53	54.1%	45	45.9%		
Gender	Male	48	72.7%	18	27.3%	5.23 (1)	0.022
	Female	40	52.6%	36	47.4%		
Age range	0–5 years	19	63.3%	11	36.7%	2.14 (2)	0.342
	6–10 years	37	68.5%	17	31.5%		
	11–16 years	32	55.2%	26	44.8%		
BMI range	Normal weight	60	69.8%	26	30.2%	10.58 (3)	0.014
	Overweight	20	47.6%	22	52.4%		
	Risk of obesity	7	77.8%	2	22.2%		
	Obesity	1	20.0%	4	80.0%		
Vaccination	Not vaccinated	38	52.8%	34	47.2%	12.05 (2)	0.002
	Complete cycle	46	78.0%	13	22.0%		
	Incomplete cycle	4	36.4%	7	63.6%		
COVID-19 severity	Non severe	76	76.0%	24	24.0%	26.26 (1)	<0.001
	Severe	12	28.6%	30	71.4%		

Percentages are row percentages. Pearson’s chi-square test was used unless otherwise specified.

**Table 5:** Logistic regression analysis of factors associated with Long COVID among SARS-CoV-2-infected children (COVID = 1; *n* = 142).

Variable	Category	Crude OR		*p*-Value	Adjusted OR		*p*-Value
		Value	95% CI		Value	95% CI	
Allergy	Allergic vs. Non-allergic	3.30	1.39–7.82	0.007	3.12	1.20–8.09	0.019
Sex	Female vs. Male	2.40	1.14–5.05	0.022	2.18	0.97–4.91	0.058
Age group	6–10 vs. 0–5 years	0.79	0.31–2.03	0.629	0.83	0.30–2.30	0.722
	11–16 vs. 0–5 years	1.40	0.57–3.47	0.463	1.36	0.48–3.88	0.568
BMI range	Overweight vs. Normal Weight	2.54	1.14–5.64	0.022	2.10	0.88–5.04	0.094
	Risk of obesity vs. Normal	0.66	0.13–3.35	0.619	0.61	0.11–3.41	0.573
	Obesity vs. Normal	9.23	0.96–88.55	0.054	6.48	0.62–67.37	0.120
Vaccination	Complete vaccination vs. no vaccination	0.34	0.15–0.77	0.009	0.54	0.16–1.83	0.323
	Incomplete vs. Not Vaccinated	1.96	0.54–7.13	0.303	1.72	0.42–7.02	0.452
COVID-19 severity	Severe vs. non-severe COVID-19	7.92	3.35–18.72	<0.001	6.84	2.72–17.21	<0.001

The adjusted model includes allergic status, sex, age group, BMI category, vaccination status and COVID-19 severity. BMI: Body Mass Index; CI: confidence interval; OR: odds ratio.

**Table 6:** Multinomial logistic regression analysis of pharyngitis etiology after removal of COVID-19 restrictions.

Predictor	GAS Pharyngitis OR		*p*-Value	VIRAL Pharyngitis OR		*p*-Value
	Value	95% CI		Value	95% CI	
Allergy	0.71	0.39–1.29	0.265	2.07	0.67–6.36	0.290
Age 6–10 vs. 0–5	0.84	0.36–1.94	0.680	0.92	0.18–4.58	0.920
Age 11–16 vs. 0–5	0.68	0.29–1.63	0.400	0.77	0.15–3.94	0.750
Female gender	1.39	0.79–2.46	0.250	1.18	0.46–3.04	0.730

Reference category: no pharyngitis. Models were adjusted for age group and sex.

## Data Availability

The data supporting the findings of this study are available from the corresponding author upon reasonable request, subject to ethical and privacy restrictions. The questionnaire used in this study is provided in the Supplementary Materials.

## Supplementary Materials

The following supporting information can be downloaded at: https://www.mdpi.com/article/10.3390/jcm15103982/s1, Supplementary File S1: Study Questionnaire; Table S1: Sensitivity analyses for Long COVID outcome; Table S2: Model calibration and goodness-of-fit.
