# Supplementary material for: Allergic Status, Long COVID, and Post-Restriction Respiratory Outcomes in Children: A Single-Center Questionnaire-Based Study
Source: J Clin Med. 2026 May 21;15(10):3982. doi: 10.3390/jcm15103982 (PMC13206905; doi:10.3390/jcm15103982)
Supplement: Supplementary file 1 [file jcm-15-03982-s001.zip › jcm-4260324-supplementary.pdf]

## SUPPLEMENTARY FILE S1. Study Questionnaire

Instructions: This questionnaire was completed by parents or legal guardians. Please select the most appropriate answer or fill in the requested information.

### SECTION 1. DEMOGRAPHIC DATA

1. Sex of the child:

□ Male

□ Female

2. Age (years): ________

### SECTION 2. COVID-19 HISTORY

3. Has the child received COVID-19 vaccination?

□ No

□ Yes → Date(s): ____________

4. Has the child had COVID-19 infection?

□ No

□ Yes

If yes:
- Date of positive test: ____________
- Date of negativization: ____________

### SECTION 3. ACUTE COVID-19 CLINICAL PRESENTATION

5. Clinical course of COVID-19 infection:

□ Asymptomatic

□ Symptomatic

If symptomatic, indicate all that apply:

□ Rhinitis

☐ Conjunctivitis

☐ Fever

☐ Cough

☐ Sore throat

☐ Asthenia/weakness

☐ Anosmia/hyposmia

☐ Ageusia

☐ Dysgeusia

☐ Myalgia

☐ Pneumonia

☐ Headache

☐ Eye Pain

☐ Dyspnea

☐ Chills

☐ Vomiting

☐ Diarrhea

☐ Skin rash

6. Was hospitalization required?

☐ No

☐ Yes → Specify: ____________

## SECTION 4. POST-COVID (LONG COVID) SYMPTOMS

7. After negativization, did the child experience persistent symptoms compatible with Long COVID (condition occurring in individuals with a history of confirmed SARS-CoV-2 infection, typically 3 months after disease onset, with symptoms lasting at least 2 months and not attributable to alternative diagnoses)?

□ No

□ Yes

If yes, indicate all that apply:

□ Persistent fatigue

□ Rhinitis

□ Fever

□ Muscle weakness

□ Asthenia

□ Diffuse pain

□ Muscle pain

□ Reduced appetite

□ Dyspnea

□ Persistent cough

□ Chest pain

□ Palpitations

□ Headache

□ Tinnitus

□ Memory difficulties

□ Concentration problems

□ Anosmia/hyposmia/parosmia

□ Disgeusia

□ Taste alterations

□ Nausea/vomiting

□ Abdominal pain

☐ Diarrhea

☐ Rash

☐ Alopecia

☐ Irritability

☐ Anxiety

☐ Depression

☐ Sleep disturbances

## SECTION 5. ALLERGIC DISEASES

8. Does the child have any of the following conditions?

☐ Allergic rhinitis

☐ Asthma

☐ Both rhinitis and asthma

☐ Food allergy

☐ Atopic dermatitis

☐ Other: ____________

☐ None

## SECTION 6. ALLERGEN POSITIVITY

9. Inhalant allergens (if applicable):

☐ Dust mites

☐ Grasses

☐ Molds (e.g., Alternaria)

☐ Parietaria

☐ Olive

☐ Dog epithelium

□ Cat epithelium

□ Birch

□ Cypress

□ Other pollens

□ None

10. Food allergens (if applicable):

□ Milk

□ Egg

□ Nuts

□ Peanut

□ Fish/shellfish

□ Soy

□ Wheat

□ Other: ___________

□ None

## SECTION 7. ALLERGIC SYMPTOM EVOLUTION AFTER PANDEMIC

11. Compared to previous years with COVID-19 restrictions, allergic rhinitis symptoms are:

□ Worsened

□ Stable

□ Improved

12. Compared to previous years with COVID-19 restrictions, asthma symptoms are:

□ Worsened

□ Stable

□ Improved

13. Degree of change in  allergic rhinitis symptoms:

□ None

□ Mild

□ Moderate

□ Severe

14. Degree of change in asthma symptoms:

□ None

□ Mild

□ Moderate

□ Severe

## SECTION 8. ALLERGIC TREATMENT

15. Use of inhaled corticosteroid therapy:

□ Not used

□ This year

□ Previous year

□ Both years

16. Use of bronchodilator therapy:

□ Not used

□ Same frequency

□ More frequent

□ Less frequent

17. Use of nasal corticosteroid therapy :

□ Not used

□ Same frequency

□ More frequent

□ Less frequent

18. Use of antihistamines:

□ Not used

□ Same frequency

□ More frequent

□ Less frequent

## SECTION 9. BACTERIAL OR VIRAL PHARYNGITIS AFTER PANDEMIC

19. After the pandemic, did the child suffer from bacterial pharyngitis documented by a doctor based on the suggestive clinical symptoms and the positive strep result of the throat swab?

□ Yes
□ No

20. How often, compared to the pre-pandemic period?

□ Same frequency
□ More frequent
□ Less frequent

21. After the pandemic, did the child suffer from viral pharyngitis documented by a doctor based on the suggestive clinical symptoms and the negative strep result of the throat swab?

□ Yes
□ No

22. How often, compared to the pre-pandemic period?

□ Same frequency
□ More frequent
□ Less frequent

## Supplementary Appendix

This appendix provides detailed results of additional analyses performed after peer review, including sensitivity analyses and model calibration assessment.

### Supplementary Sensitivity Analyses

To further evaluate the robustness of the findings, a comprehensive set of sensitivity analyses was performed to evaluate the robustness of the association between allergic status and Long COVID, accounting for potential outcome misclassification, sparse categories, vaccination coding, and age-related confounding.

- Exclusion of respiratory-overlapping symptoms (rhinitis, cough, anosmia, exertional dyspnea) did not materially change Long COVID prevalence (38.0%), and allergic status remained independently associated (aOR ≈ 4.47).
- A more conservative Long COVID definition excluding additional non-specific symptoms confirmed persistence of the association (aOR 3.84, 95% CI 1.30–11.36; p = 0.015).
- Collapsing BMI into normal weight versus excess weight improved model stability, with excess weight remaining associated with Long COVID (aOR 4.24, 95% CI 1.64–10.98; p = 0.0030).
- Simplified vaccination coding showed no independent association with Long COVID after adjustment.
- Age-adjusted and stratified analyses confirmed persistence of the association, with exploratory evidence suggesting possible age-related heterogeneity (exploratory finding).
- Restriction to vaccine-eligible children (≥5 years) confirmed strong protection of vaccination against infection (aOR 0.145, p < 0.001).

### Table S1. Sensitivity analyses for Long COVID outcome

| Analysis | Aim | Main result | Interpretation |
|---|---|---|---|
| Respiratory-overlap symptoms excluded | Reduce symptom overlap with allergic respiratory disease | Long COVID prevalence unchanged (38.0%); allergy aOR 4.47 (95% CI 1.55–12.94), p=0.0057 | Main finding robust |
| Conservative Long COVID definition | Reduce outcome misclassification | Allergy aOR 3.84 (95% CI 1.30–11.36), p=0.015 | Association persists |

| | | | |
|---|---|---|---|
| BMI recoding | Reduce sparse-data instability | Excess weight aOR 4.24 (95% CI 1.64–10.98), p=0.0030 | More stable estimate |
| Simplified vaccination coding | Improve robustness | No independent association with Long COVID | Avoids overclaim |
| Age adjustment/ stratification | Control confounding | Association persisted; stronger in 11–16 years | Exploratory effect |

## Model calibration assessment

Goodness-of-fit of the main logistic regression models was assessed using the Hosmer–Lemeshow test.
The original multivariable Long Covid model showed suboptimal calibration ($\chi^2 = 22.34$, df = 8, $p = 0.004$), likely reflecting model complexity and sparse categories.
By contrast, the revised sensitivity model (conservative Long Covid definition, collapsed BMI categories, simplified vaccination coding) showed good calibration ($\chi^2 = 5.85$, df = 8, $p = 0.664$).
The infection model restricted to vaccine-eligible children showed acceptable calibration ($\chi^2 = 15.13$, df = 8, $p = 0.057$).
These findings support the parsimonious sensitivity models as the most robust inferential framework in the revised manuscript.

## Table S2. Model calibration and goodness-of-fit

| Model | N | HL $\chi^2$ | df | p-value | Interpretation |
|---|---|---|---|---|---|
| Original Long COVID model | 142 | 22.34 | 8 | 0.004 | Suboptimal calibration |
| Sensitivity Long COVID model | 142 | 5.85 | 8 | 0.664 | Good calibration |
| Infection model (eligible children) | 177 | 15.13 | 8 | 0.057 | Acceptable calibration |

## Supplementary File S1. Study Questionnaire is provided separately.
